# KAN-GIN: Adaptive Nonlinear Molecular Representation Learning for Drug-Target Affinity Prediction

**DOI:** 10.3390/ph19091458

**Published:** 2026-09-14

**Authors:** Abla Bedoui, Nithyadevi Duraisamy, Mohammed Cherkaoui

**Affiliations:** School of Computer Science, Digital Engineering and AI, Long Island University, Brooklyn, NY 11201, USA; nithyadevi.duraisamy@liu.edu (N.D.); mohammed.cherkaoui@liu.edu (M.C.)

**Keywords:** drug-target affinity prediction, graph neural networks, Kolmogorov-Arnold Networks, molecular representation learning, drug repurposing, artificial intelligence in drug discovery

## Abstract

**Background/Objectives:** Accurate drug-target affinity (DTA) prediction supports virtual screening, lead optimization, and drug repurposing. This study investigates whether replacing the conventional post-aggregation multilayer perceptron within a Graph Isomorphism Network drug encoder with a Kolmogorov-Arnold Network (KAN) transformation improves DTA prediction. **Methods:** The resulting KAN-GIN framework combines molecular-graph representations with a convolutional protein-sequence encoder. It was evaluated on the DAVIS, KIBA, and METZ benchmarks against a matched GIN baseline and representative multiscale and graph-pretraining-based DTA architectures under a common split and evaluation protocol. Complementary analyses examined latent-space organization, gradient-based molecular attribution, and an enhancer of zeste homolog 2 (EZH2) candidate-ranking case study. **Results:** KAN-GIN consistently reduced prediction error relative to the matched GIN baseline and achieved competitive performance against the additional architectures, including the strongest overall results on METZ. The latent-space findings were dataset-dependent, while gradient-based attribution identified molecular substructures contributing to individual predictions. The EZH2 case study illustrated how KAN-GIN can support candidate ranking before structure-based evaluation. **Conclusions:** KAN-based post-aggregation transformations represent an emerging design option for graph-based DTA prediction. However, the latent-space and structure-based findings remain computational and should not be interpreted as evidence of mechanistic biological relevance without prospective experimental validation.

## 1. Introduction

Drug-target affinity (DTA) prediction estimates the strength of interaction between small-molecule compounds and biological targets. Accurate computational prediction can support virtual screening, lead optimization, drug repurposing, and candidate prioritization while reducing the cost and time associated with large-scale experimental screening [1,2,3]. Developing accurate and generalizable DTA models has therefore become an important objective in computational drug discovery.

Deep learning has shifted DTA prediction from handcrafted descriptors toward end-to-end representation learning. Graph neural networks (GNNs) are particularly suitable for molecular modeling because they represent atoms as nodes and chemical bonds as edges, enabling molecular topology to be learned directly from graph structure. Graph-based DTA approaches combine learned molecular representations with protein-sequence encoders to predict continuous affinity values [4]. Recent DTA architectures have further incorporated multiscale graph learning, attention mechanisms, Transformer-based architectures, and pretrained molecular and protein representations, highlighting the growing diversity of representation-learning strategies for affinity prediction.

Despite these advances, many message-passing architectures continue to use conventional multilayer perceptrons (MLPs) to transform node representations after neighborhood aggregation. This motivates closer examination of the transformation operator itself as a distinct component of graph-based DTA architectures. Kolmogorov-Arnold Networks (KANs) provide an alternative functional approximation mechanism in which network edges are parameterized by learnable univariate functions rather than relying exclusively on fixed node-wise activation functions [5]. Although KAN modules have recently been incorporated into graph-learning architectures [6,7], controlled evaluation of a KAN-based post-aggregation transformation specifically within multimodal DTA prediction remains limited.

In this work, we develop KAN-GIN, a DTA framework that replaces the conventional post-aggregation MLP within a GIN drug encoder with a KAN-based functional approximator. The surrounding drug-protein prediction framework is preserved to support a focused evaluation of the transformation operator. Drug compounds are represented as molecular graphs, target proteins are encoded from their amino-acid sequences, and the resulting representations are fused to predict binding affinity. We hypothesize that the adaptive nonlinear transformation may improve affinity prediction and produce learned representations that are more strongly organized with respect to benchmark affinity labels.

KAN-GIN is evaluated on the DAVIS, KIBA, and METZ benchmark datasets. In addition to predictive performance, this study examines latent representation organization, molecular-complexity-dependent performance, and gradient-based molecular attribution. An EZH2 drug-repurposing case study is further presented as a computational illustration of how DTA prediction can be incorporated into candidate prioritization before complementary structure-based evaluation.

The principal contributions of this study are as follows:

- We adapt KAN-based graph transformations to multimodal DTA prediction by replacing the conventional post-aggregation MLP within a GIN drug encoder while preserving the surrounding drug-protein prediction framework.
- We systematically evaluate KAN-GIN across the DAVIS, KIBA, and METZ benchmarks, including comparison with a matched GIN baseline and representative recent DTA architectures, and examine its predictive behavior under both random-interaction and entity-level generalization settings.
- We complement predictive evaluation with affinity-label-associated latent-representation analyses, molecular-complexity analysis, gradient-based molecular attribution, and an EZH2 candidate-prioritization case study.

## 2. Related Work

### 2.1. Drug-Target Affinity Prediction

DTA prediction estimates the strength of interaction between a drug compound and a biological target. Early computational approaches primarily relied on similarity-based learning and handcrafted molecular descriptors. Representative methods such as KronRLS [8] and SimBoost [9] used drug and protein similarity matrices, as well as manually engineered features, to predict binding affinity. Additional machine-learning approaches, including kernel methods and matrix factorization techniques, also demonstrated competitive performance on early benchmark datasets [10].

The emergence of deep learning shifted DTA prediction toward end-to-end representation learning. DeepDTA [11] represented drugs as SMILES strings and proteins as amino-acid sequences and learned latent representations directly from raw inputs using convolutional neural networks. WideDTA [12] further extended sequence-based learning by incorporating additional sequence patterns and domain-specific information. Subsequent methods, including DeepAffinity [13] and DeepConv-DTI [14], further demonstrated the potential of deep representation learning for affinity prediction.

More recent DTA approaches have increasingly combined heterogeneous molecular and protein representations. DTBAffinity integrates physicochemical and topological ligand descriptors, structural and circular molecular fingerprints, protein-sequence descriptors, and pretrained ESM embeddings within a regularized gradient-boosting framework [15]. Such approaches illustrate the broader trend toward combining complementary handcrafted, learned, and pretrained representations for DTA prediction.

### 2.2. Graph Neural Networks for Molecular Representation Learning

GraphDTA [4] was among the first frameworks to demonstrate the advantages of graph-based molecular encoders for affinity prediction by integrating GCN [16], GAT [17], GAT-GCN, and GIN [18] architectures with sequence-based protein encoders. These results highlight the value of explicitly representing molecular topology rather than treating compounds solely as linear molecular sequences.

Subsequent studies extended graph-based DTA prediction through multiscale representations, attention mechanisms, interaction-aware architectures, and alternative graph-learning strategies. MGraphDTA [19] introduced multiscale graph feature extraction to capture molecular information at different representation levels, while DGraphDTA [20] and BridgeDPI [21] explored graph-based and interaction-aware mechanisms for modeling drug-target relationships.

More recently, pretrained molecular representations have become increasingly important in molecular learning. Graph transformers [22] and pretrained molecular models such as GROVER [23], MolCLR [24], GraphMVP [25], and GEM [26] have demonstrated the potential of large-scale pretraining to improve molecular representation learning and transfer across downstream tasks. In DTA prediction, GNPDTA [27] incorporates pretrained graph representations into the drug-target prediction framework, providing a representative example of this emerging direction.

These developments have primarily focused on improving molecular representations through graph architecture design, multiscale feature extraction, interaction modeling, or pretraining. The present study examines a complementary architectural question: whether the nonlinear transformation applied after neighborhood aggregation can itself be modified to improve graph-based DTA prediction.

### 2.3. Kolmogorov-Arnold Networks

Kolmogorov-Arnold Networks (KANs) are neural architectures inspired by the Kolmogorov-Arnold representation theorem [5]. Whereas conventional MLPs generally use scalar weights on network edges and fixed nonlinear activation functions at their nodes, KANs parameterize edges using learnable univariate functions. These functions can be represented using B-splines, radial basis functions, Fourier series, or other functional bases, providing an adaptive mechanism for approximating nonlinear mappings. Several variants, including FastKAN and efficient-KAN, have been proposed to reduce the computational cost of the original spline-based formulation.

The integration of KAN modules into graph neural networks has already been investigated in several settings. Bresson et al. [6] introduced KAN-based graph layers inspired by GCN, GAT, and GIN architectures and evaluated both B-spline- and radial-basis-function-based implementations. Li et al. [7] further investigated KAN application to node embedding, message passing, and graph-level readout for molecular property prediction. These studies show that KAN-based graph transformations constitute an established but still emerging research direction.

More recently, Rakib et al. introduced KANPM-DTA, a multimodal DTA framework combining pretrained molecular and protein representations, graph-based encoders, feature-fusion mechanisms, and a KAN-based affinity-regression head [28]. Specifically, KANPM-DTA employs ESM-C protein embeddings, ESM-2-derived protein contact maps, ChemBERTa-2 molecular embeddings, GCN-GAT graph encoders, gated fusion, and linear attention before applying a KAN block to the fused representation for affinity prediction. This work demonstrates the potential of incorporating KANs into a more extensive pretrained multimodal DTA architecture.

Although KANPM-DTA and the present framework both exploit the nonlinear approximation capacity of KANs, they introduce the KAN component at different stages of the prediction pipeline. KANPM-DTA applies a KAN as the final regression head after multimodal feature encoding and fusion. In contrast, KAN-GIN integrates FastKAN transformations directly in the molecular graph representation-learning process by replacing the conventional post-aggregation MLP within the GIN layers, while retaining a convolutional protein-sequence encoder and a conventional prediction head. This controlled design isolates the effect of the nonlinear graph-transformation mechanism from broader changes involving external pretraining, protein encoding, feature fusion, and affinity regression.

### 2.4. Interpretability in Molecular Machine Learning

Numerous interpretability techniques have been proposed for molecular machine learning, including attention visualization [17], explainable graph neural networks [29], feature attribution methods [30], gradient-based saliency analysis [31], and graph explanation frameworks [32]. Among these approaches, atom-level saliency maps offer an intuitive mechanism for identifying the atoms and functional groups that contribute most strongly to a prediction, enabling researchers to relate learned representations to chemically relevant pharmacophoric motifs.

## 3. Results

### 3.1. Overall Predictive Performance

Table 1 compares KAN-GIN with the matched GIN baseline and two representative DTA architectures. Relative to GIN, KAN-GIN reduced MSE from 0.2622 to 0.2435 on DAVIS, from 0.2342 to 0.2163 on KIBA, and from 0.3594 to 0.3155 on METZ, corresponding to reductions of approximately 7.1%, 7.6%, and 12.2%, respectively. KAN-GIN also improved RMSE, MAE, $R^{2}$, and CI relative to the matched GIN baseline on all three datasets. Performance relative to the additional architectures was dataset-dependent. On DAVIS, GNPDTA obtained the lowest MSE and RMSE and the highest $R^{2}$, whereas KAN-GIN obtained the lowest MAE and highest CI. MGraphDTA achieved the strongest overall performance on KIBA. On METZ, KAN-GIN achieved the best mean value for all five reported metrics. These findings show that KAN-GIN consistently improves upon its directly matched GIN baseline while remaining competitive with the representative multiscale and pretrained graph-based DTA models. However, no single architecture was superior across all datasets. The results therefore support the utility of KAN-based post-aggregation transformations without implying universal state-of-the-art performance.

### 3.2. Controlled Ablation of the KAN Transformation

To isolate the contribution of the KAN-based nonlinear transformation, we performed a controlled ablation in which the conventional GIN transformation and the KAN-based variant were evaluated under identical experimental conditions. Both variants used the same molecular and protein inputs, graph message-passing structure, protein encoder, fusion and prediction components, data partitions, and optimization settings. The only intended experimental change was the nonlinear transformation applied within the molecular graph encoder. Results were averaged over three independent runs with random seeds 42, 123, and 2025, and the checkpoint with the minimum validation MSE was evaluated on the held-out test set.

As shown in Table 2, the effect of the KAN-based transformation was dataset-dependent. On DAVIS, KAN-GIN reduced the mean MSE from 0.2554±0.0081 to 0.2467±0.0048 and increased $R^{2}$ from 0.6730±0.0087 to 0.6840±0.0141. The MAE also decreased from 0.3055±0.0124 to 0.2918±0.0136. However, the concordance index did not improve, decreasing from 0.8811±0.0040 for GIN to 0.8756±0.0088 for KAN-GIN. Thus, the improvement on DAVIS was observed primarily in regression error rather than ranking performance. A clearer improvement was observed on METZ. KAN-GIN reduced the mean MSE from 0.3459±0.0133 to 0.3196±0.0104 and the MAE from 0.4477±0.0065 to 0.4288±0.0055, while increasing $R^{2}$ from 0.6214±0.0079 to 0.6501±0.0043. CI also increased slightly, from 0.7935±0.0037 to 0.7983±0.0022. On KIBA, KAN-GIN similarly reduced the mean MSE from 0.2259 to 0.2135, while $R^{2}$ increased from 0.6782 to 0.6969 and CI from 0.8289 to 0.8349. Overall, these controlled experiments indicate that replacing the conventional GIN transformation with the KAN-based transformation can improve affinity-regression accuracy, but the magnitude and consistency of the improvement depend on the dataset and evaluation metric. These results therefore support a more limited claim of dataset-dependent predictive benefit rather than a uniform improvement across all metrics.

### 3.3. Generalization to Unseen Drugs and Targets

Generalization performance under cold-drug, cold-target, and double-cold evaluation is summarized in Appendix A (Table A2). KAN-GIN achieved a lower mean MSE than GIN in 11 of the 12 dataset-scenario combinations, with the most consistent improvements observed under the cold-drug protocol. Relative to GIN, KAN-GIN reduced cold-drug MSE by 14.1% on DAVIS, 15.3% on KIBA, and 11.4% on METZ. These improvements were observed across all three training seeds for each dataset, indicating that the reduction was not attributable to a single initialization.

Under the more restrictive double-cold protocol, KAN-GIN reduced the mean MSE by 10.2% on DAVIS, 9.8% on KIBA, and 22.0% on METZ. Cold-target behavior was more dataset-dependent: KAN-GIN reduced MSE by 11.5% on KIBA and 18.1% on METZ, whereas DAVIS showed essentially comparable performance, with MSE values of 0.6240±0.0291 for GIN and 0.6266±0.0602 for KAN-GIN.

Across the complete generalization experiment, KAN-GIN improved the mean $R^{2}$ in 11 of the 12 settings, while MAE and CI exhibited more scenario-dependent differences. Thus, the results indicate generally improved error-based generalization, particularly for unseen drugs, without suggesting uniform superiority across every dataset, split, and evaluation metric.

### 3.4. Affinity-Aware Latent Representation Organization

We next examined whether the predictive gains of KAN-GIN were accompanied by more structured molecular representations. For each interaction in the held-out test set, a joint drug-target representation was extracted from the penultimate fusion layer after combining the molecular graph and protein sequence representations. Consequently, every latent vector corresponded to one drug-target pair and its experimentally measured affinity.

#### 3.4.1. Affinity-Bin Separability

**Figure 1:**
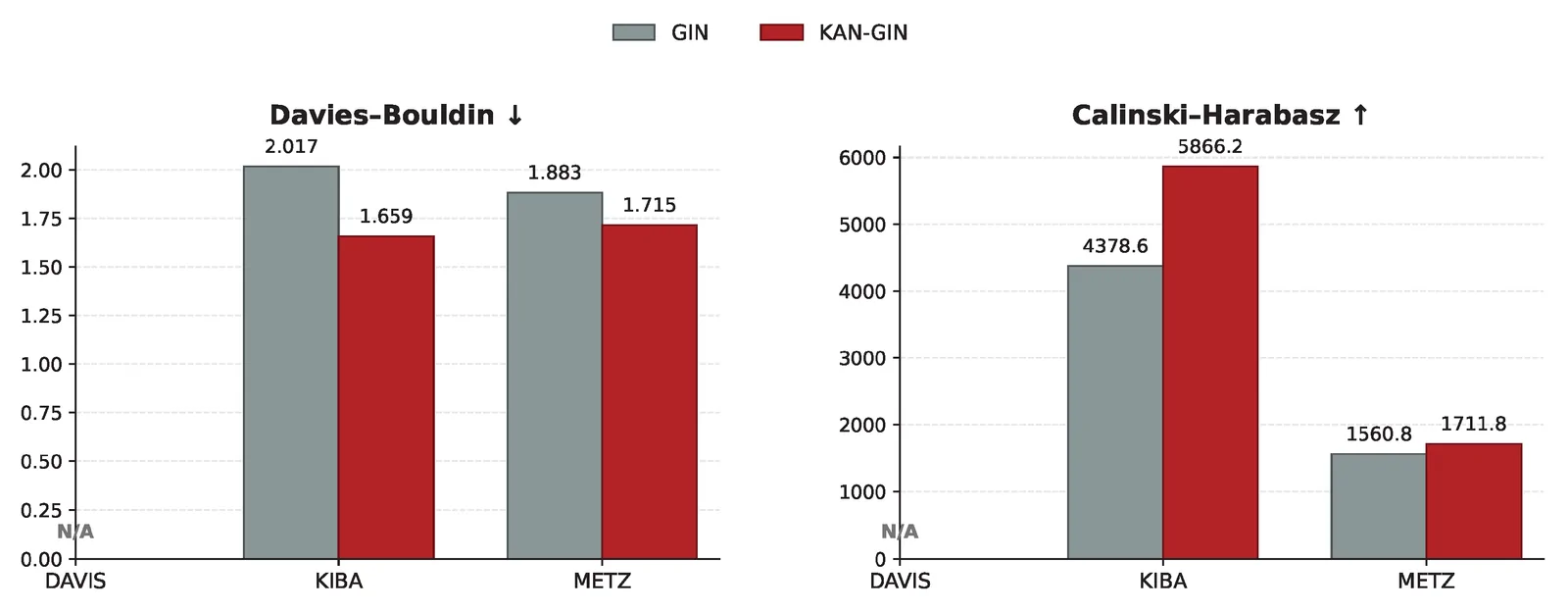
Affinity-associated organization of joint drug-target representations. (**Left**) Davies-Bouldin index, where lower values indicate greater compactness and separation of the predefined affinity groups. (**Right**) Calinski-Harabasz index, where higher values indicate greater between-group relative to within-group dispersion. The downward and upward arrows indicate that lower Davies-Bouldin values and higher Calinski-Harabasz values, respectively, represent better affinity-group organization. KAN-GIN improved both measures on KIBA and METZ. DAVIS is reported as N/A because valid affinity groups could not be obtained under the predefined quantile-binning procedure. The indices should be compared between models within each dataset and not across datasets.

To assess whether affinity-related information is more clearly organized in latent space, we evaluated embedding separability using the Davies-Bouldin (DB) and Calinski-Harabasz (CH) indices. As shown in Figure 1, KAN-GIN improved both indices on KIBA and METZ. On KIBA, the DB score decreased from 2.017 to 1.659, while the CH score increased from 4378.6 to 5866.2. On METZ, the DB score decreased from 1.883 to 1.715 and the CH score increased from 1560.8 to 1711.8. These results indicate improved global affinity-bin organization of the joint representations on KIBA and METZ.

#### 3.4.2. Local Affinity Predictability

While affinity-bin separability evaluates the global organization of affinity groups, the local analysis examines whether neighboring joint drug-target representations preserve fine-grained pair-specific affinity relationships. Local organization was evaluated using two complementary metrics described in Section 4.7: (i) drug-grouped out-of-fold kNN $R^{2}$, which measures local affinity predictability; and (ii) cross-drug Local MAD, which measures affinity consistency among neighboring representations corresponding to different compounds.

**Figure 2:**
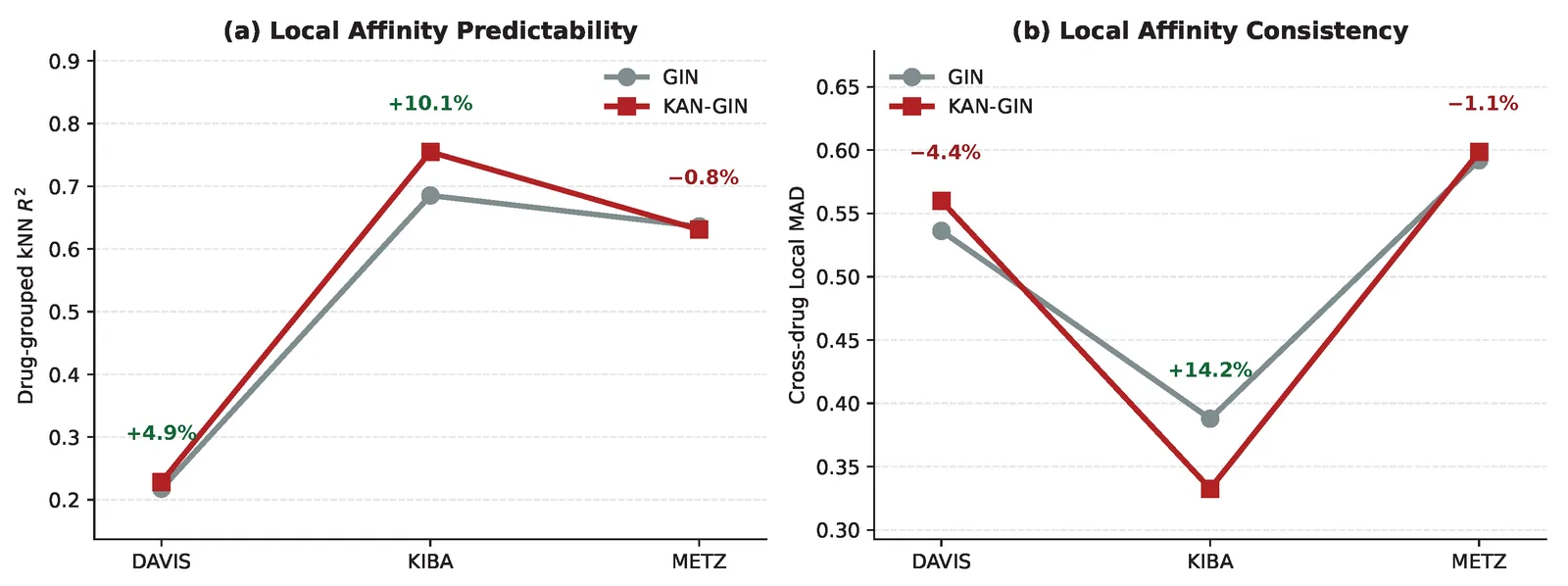
Local affinity organization of joint drug-target representations. (**a**) Drug-grouped out-of-fold kNN affinity predictability, measured using $R^{2}$, where higher values are better. (**b**) Cross-drug Local MAD, where lower values are better. The strongest and most consistent local improvement was observed on KIBA, while DAVIS and METZ showed mixed or small numerical differences.

As shown in Figure 2, the strongest and most consistent local improvement was observed on KIBA. Drug-grouped out-of-fold kNN $R^{2}$ increased from 0.6853 for GIN to 0.7547 for KAN-GIN, while cross-drug Local MAD decreased from 0.3878 to 0.3326.

These analyses show that affinity-related information is organized within the joint drug-target representation space, although the extent of this organization varies across datasets. The clearest local improvement was observed on KIBA, while the global affinity-bin analysis also favored KAN-GIN on KIBA and METZ. Such dataset-dependent behavior is consistent with differences in chemical composition, target distribution, interaction density, and affinity-label structure among the benchmark datasets. These analyses complement the end-to-end prediction results by describing the geometry of the learned representations and are not intended as standalone evidence of biological mechanisms.

### 3.5. Molecular Complexity-Dependent Performance Gains

**Figure 3:**
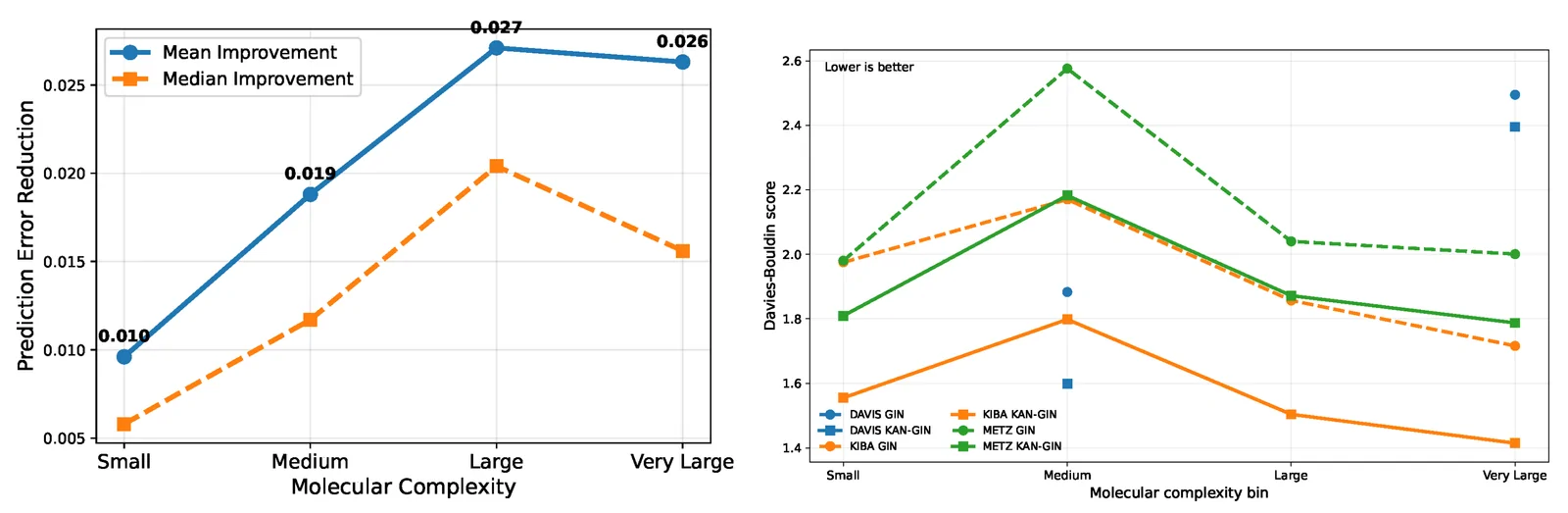
Complexity-dependent behavior of KAN-GIN. (**Left**) Mean and median pair-level prediction-error reduction achieved by KAN-GIN relative to GIN across molecular-complexity categories. Prediction error was calculated independently for every held-out drug-target pair. Positive values indicate lower absolute prediction error for KAN-GIN. (**Right**) Complexity-stratified organization of joint drug-target representations measured using the Davies-Bouldin index, where lower values indicate greater affinity-group compactness and separation. KAN-GIN produced lower DB scores across the available complexity strata, although the magnitude of the difference varied non-monotonically. DAVIS categories for which valid affinity groups could not be constructed are not reported.

To evaluate whether adaptive nonlinear transformations provide greater benefit for structurally complex compounds, molecules were grouped into complexity bins according to molecular size and structural descriptors. Improvement was defined as the reduction in absolute prediction error achieved by KAN-GIN relative to the GIN baseline. As shown in Figure 3 (left), the performance advantage of KAN-GIN increased with molecular complexity. The mean prediction error reduction increased from 0.0096 for small molecules to 0.0271 for large molecules and remained elevated for very large compounds.

The complexity-stratified latent-space component was calculated using the joint drug-target representations. As shown in Figure 3 (right), KAN-GIN produced lower DB scores than GIN across all KIBA and METZ complexity categories and across the available DAVIS categories. This indicates that the improved global affinity-bin organization was maintained across a range of molecular complexities. However, the magnitude of the DB difference was not monotonic across the complexity groups. Overall, KAN-GIN maintained better affinity-group organization across the evaluated complexity categories, indicating that its representational benefit extends to compounds with varying levels of structural complexity.

### 3.6. Saliency-Based Interpretability Analysis

**Figure 4:**
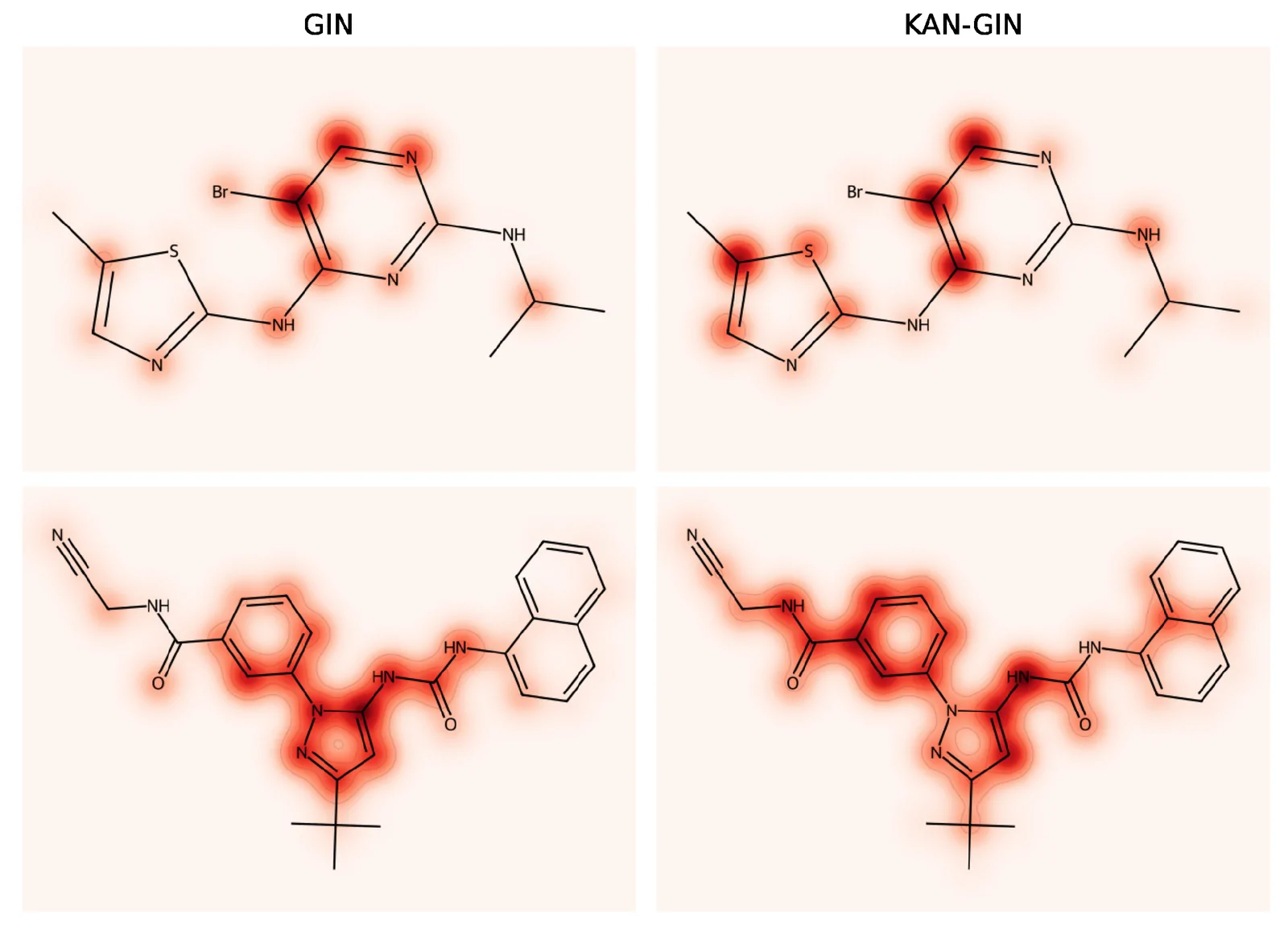
Representative atom-level saliency maps for structurally simple and complex molecules generated by GIN and KAN-GIN. Warmer colors indicate greater contribution of individual atoms to the predicted affinity score.

Figure 4 compares atom-level saliency maps generated by GIN and KAN-GIN for representative simple and structurally complex molecules. Saliency scores were computed using gradient-based attribution, where higher values indicate greater contribution of individual atoms to the predicted affinity score.

For structurally simple molecules, both models exhibit broadly similar attribution patterns. In contrast, for structurally complex compounds, KAN-GIN distributes attribution across a larger collection of interacting motifs, including aromatic systems, heterocyclic regions, and linker-associated functional groups, whereas GIN concentrates attribution within fewer localized regions. Although qualitative, these observations are consistent with the complexity-dependent performance gains shown in Figure 3, suggesting that adaptive nonlinear transformations facilitate the integration of information from multiple interacting molecular motifs.

### 3.7. EZH2 Drug-Repurposing Case Study

Following candidate standardization, pramoxine and pramoxine hydrochloride were identified as the same parent compound, leaving 10 unique candidates for the final analysis. As shown in Table 3, tirbanibulin received the highest primary docking score, followed by perphenazine and acetophenazine. The KAN-GIN models produced distinct candidate rankings across DAVIS, KIBA, and METZ, consistent with the different affinity endpoints and numerical scales represented by the three benchmark datasets.

To assess the agreement between the KAN-GIN candidate rankings and the molecular-docking results, Table 4 reports the corresponding Spearman and Kendall rank-correlation coefficients. The resulting correspondence was dataset-dependent. For the DAVIS-trained model, Spearman’s ρ=−0.612 (p=0.060) and Kendall’s τ=−0.422 (p=0.108). The KIBA-trained model showed a positive rank trend, with Spearman’s ρ=0.588 (p=0.074) and Kendall’s τ=0.467 (p=0.073), while the METZ-trained model yielded Spearman’s ρ=0.442 (p=0.200) and Kendall’s τ=0.333 (p=0.216). None of these rank correlations reached statistical significance.

Candidate-level overlap nevertheless occurred among the highest-ranked compounds. The KIBA-trained model recovered two of the three and four of the five highest-ranked docking candidates. The METZ-trained model recovered three of the docking top five, whereas the DAVIS-trained model recovered one of the docking top five. These results indicate partial, dataset-dependent overlap among the highest-priority candidates, rather than general agreement between the complete KAN-GIN and docking rankings. A training-set overlap and structural-similarity analysis of the shortlisted compounds is provided in Appendix A (Table A4).

The sensitivity analysis further showed that the observed correspondence depended on the docking-derived quantity considered. Pairwise Spearman correlations among −CDOCKER Energy, −CDOCKER Interaction Energy, and the calculated binding-energy quantity ranged from −0.152 to 0.333. The magnitude and direction of their correlations with KAN-GIN predictions also varied across scoring quantities (Table A5), and none of the model-docking correlations reached statistical significance. Thus, the different computational rankings provided complementary rather than quantitatively concordant candidate-prioritization signals.

**Figure 5:**
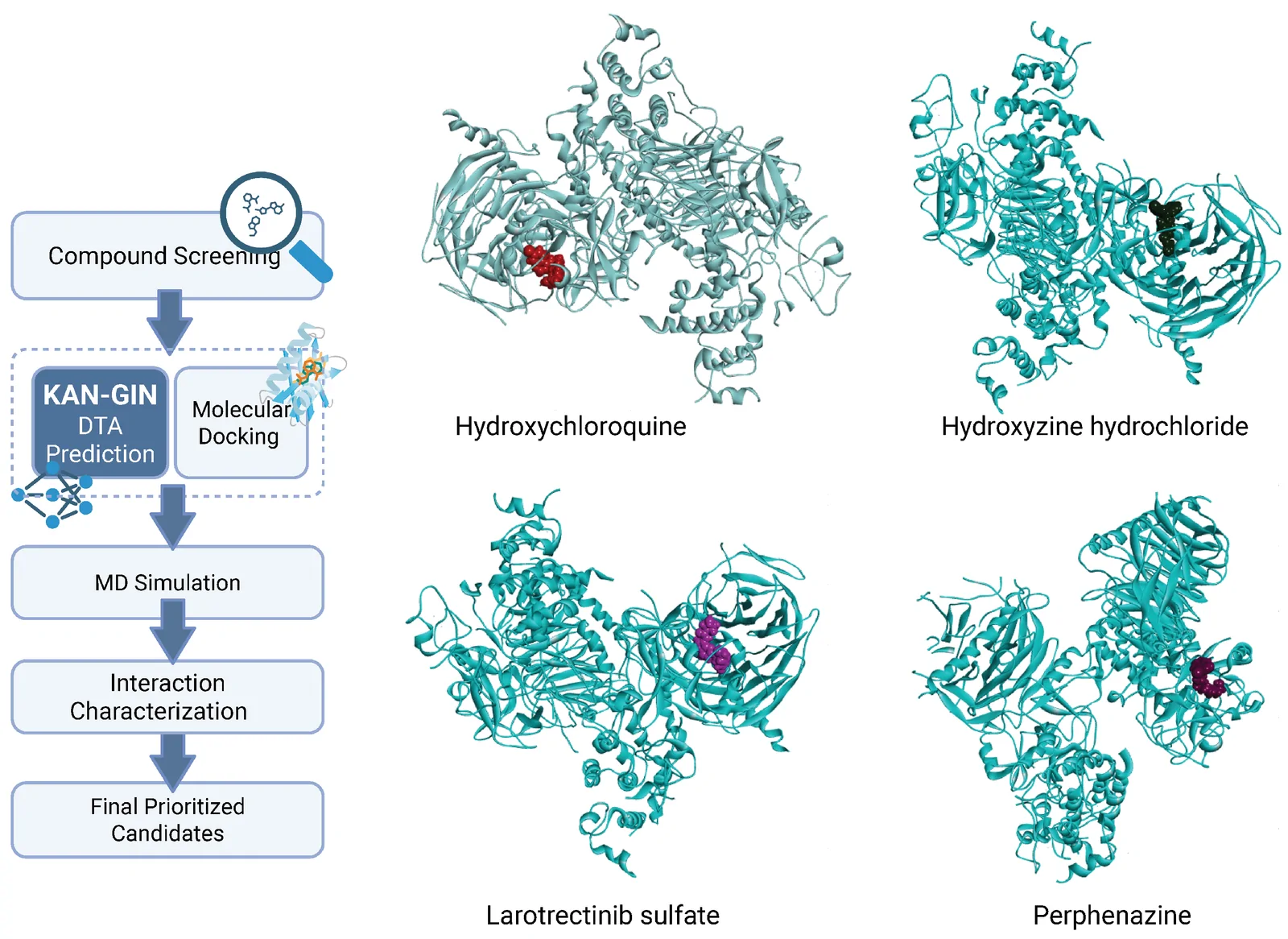
Illustrative integration of KAN-GIN into an EZH2 drug-prioritization workflow. Candidate compounds are prioritized using KAN-GIN and subsequently examined by molecular docking as an independent structure-based assessment. Representative docked protein-ligand complexes are shown for selected candidates. Molecular dynamics simulation and subsequent interaction characterization are included only to illustrate potential downstream refinement stages in a broader drug-discovery workflow; these analyses were not performed as part of the present study.

Representative docking poses for selected prioritized compounds are shown in Figure 5. The predicted complexes illustrate ligand occupation and protein-ligand interactions within the EZH2-binding region. These docking poses represent computationally predicted configurations and do not constitute experimental evidence of binding or biological activity.

## 4. Materials and Methods

### 4.1. Datasets

The proposed KAN-GIN framework was evaluated on three widely used benchmark datasets for drug-target affinity prediction: DAVIS [33], KIBA [34], and METZ [35].

The DAVIS dataset contains kinase-inhibitor binding affinities measured as dissociation constants ($K_{d}$). Following common practice in DTA prediction, the affinity values were transformed to the logarithmic $pK_{d}$ scale for model training and evaluation. The dataset contains 442 target proteins, 68 compounds, and 30,056 drug-target interactions.

The KIBA dataset integrates multiple bioactivity measurements, including $K_{i}$, $K_{d}$, and $IC_{50}$, into a unified KIBA score. After preprocessing, the dataset contains 229 proteins, 2111 compounds, and 118,254 interactions, providing a substantially larger interaction set than DAVIS.

The METZ dataset [35] contains kinase-inhibitor activity measurements involving 170 protein targets, 1423 compounds, and 35,259 drug-target interactions. Affinity values were represented on the $pK_{i}$ scale. METZ provides a complementary benchmark with a larger and more chemically diverse compound set than DAVIS.

Table 5 summarizes the principal characteristics of the three datasets. Drug and protein records were processed using a common preprocessing pipeline across the evaluated models. The specific train/validation/test partitioning strategies used for the principal benchmark, controlled ablation, and entity-level generalization experiments are described separately in Section 4.5, since these experiments were designed to address different evaluation objectives.

### 4.2. Molecular Representation

Drug compounds are represented as molecular graphs, where atoms correspond to graph nodes and chemical bonds correspond to graph edges.

Formally, a molecular graph is defined as

(1) G=(V,E),

where *V* denotes the set of atoms and *E* denotes the set of chemical bonds. For each atom, a feature vector is constructed containing chemical properties including atom type, degree, valence, aromaticity, and hydrogen count. These node features provide the initial representation used by the graph neural network. Representing molecules as graphs preserves important structural information that is not explicitly captured by linear SMILES sequences, including local neighborhoods, ring structures, branching patterns, and atom connectivity. Molecular graph construction and molecular descriptor calculation were performed using RDKit v2026.03.6.

### 4.3. Protein Representation

Target proteins are represented using their amino-acid sequences. Given a protein sequence

(2) $$P=(a_{1},a_{2},\ldots ,a_{n}),$$

each amino acid is first mapped into a learnable embedding space. The resulting embedding vectors are then processed using one-dimensional convolutional layers to capture local sequence patterns that may be informative for affinity prediction. A global pooling operation is subsequently applied to obtain a fixed-length protein embedding vector.

### 4.4. Proposed KAN-GIN Architecture

**Figure 6:**
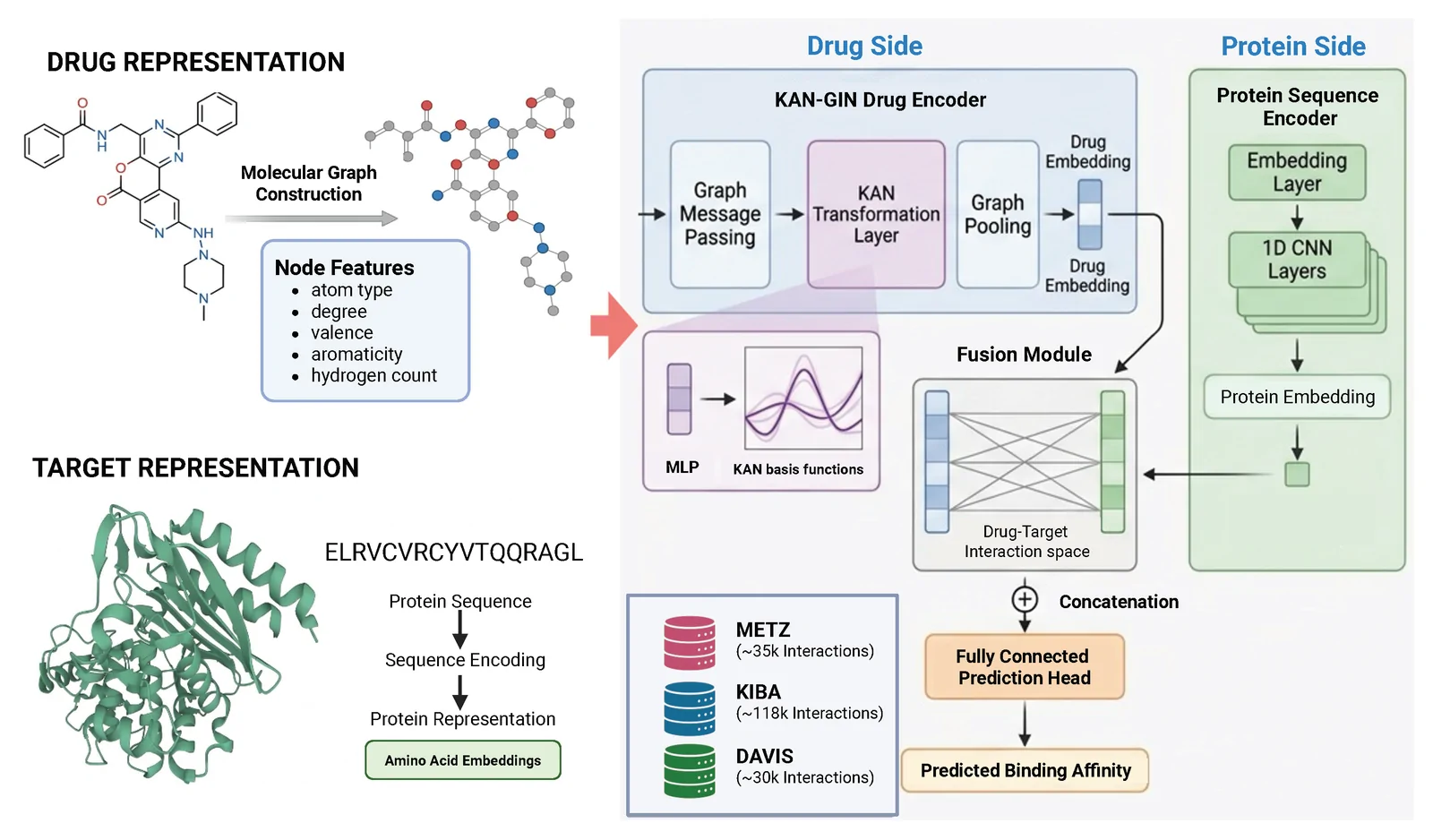
Overview of the proposed KAN-GIN architecture. Molecular graphs are processed using GIN message passing, with the conventional post-aggregation MLP transformation replaced by a KAN-based functional approximator. Protein sequences are encoded using a convolutional neural network, and the resulting molecular and protein representations are fused to predict drug-target affinity. The surrounding drug-protein prediction framework is preserved to support a focused evaluation of the nonlinear transformation mechanism.

The proposed KAN-GIN framework consists of four major components: (i) molecular graph representation, (ii) KAN-based graph message passing, (iii) protein sequence encoding, and (iv) multimodal affinity prediction. The overall architecture is illustrated in Figure 6.

#### 4.4.1. GIN-Based Message Passing

Given a molecular graph *G*, node representations are updated through neighborhood aggregation. Following the Graph Isomorphism Network (GIN) framework [18], each node combines its own representation with information aggregated from neighboring atoms before applying a nonlinear transformation. This message-passing paradigm has been shown to possess strong representational power and has become a widely used approach for molecular graph representation learning in drug discovery applications [4,18].

#### 4.4.2. Kolmogorov-Arnold Functional Approximation

A key objective of this work is to analyze the role of nonlinear transformation mechanisms in graph representation learning for DTA prediction. While conventional Graph Isomorphism Networks employ multilayer perceptrons to transform aggregated neighborhood information, the proposed framework utilizes Kolmogorov-Arnold Networks (KANs) as a more flexible functional approximation mechanism. Unlike conventional neural transformations that rely primarily on fixed activation functions, KANs model nonlinear relationships through learnable univariate functions. Given the aggregated neighborhood representation, the node update function is expressed as

(3) $$h_{v}^{\left(k\right)}={\mathrm{KAN}}^{\left(k\right)}\left(\left(1+\epsilon^{\left(k\right)}\right)h_{v}^{\left(k-1\right)}+\sum_{u\in N(v)}h_{u}^{\left(k-1\right)}\right).$$

**Figure 7:**
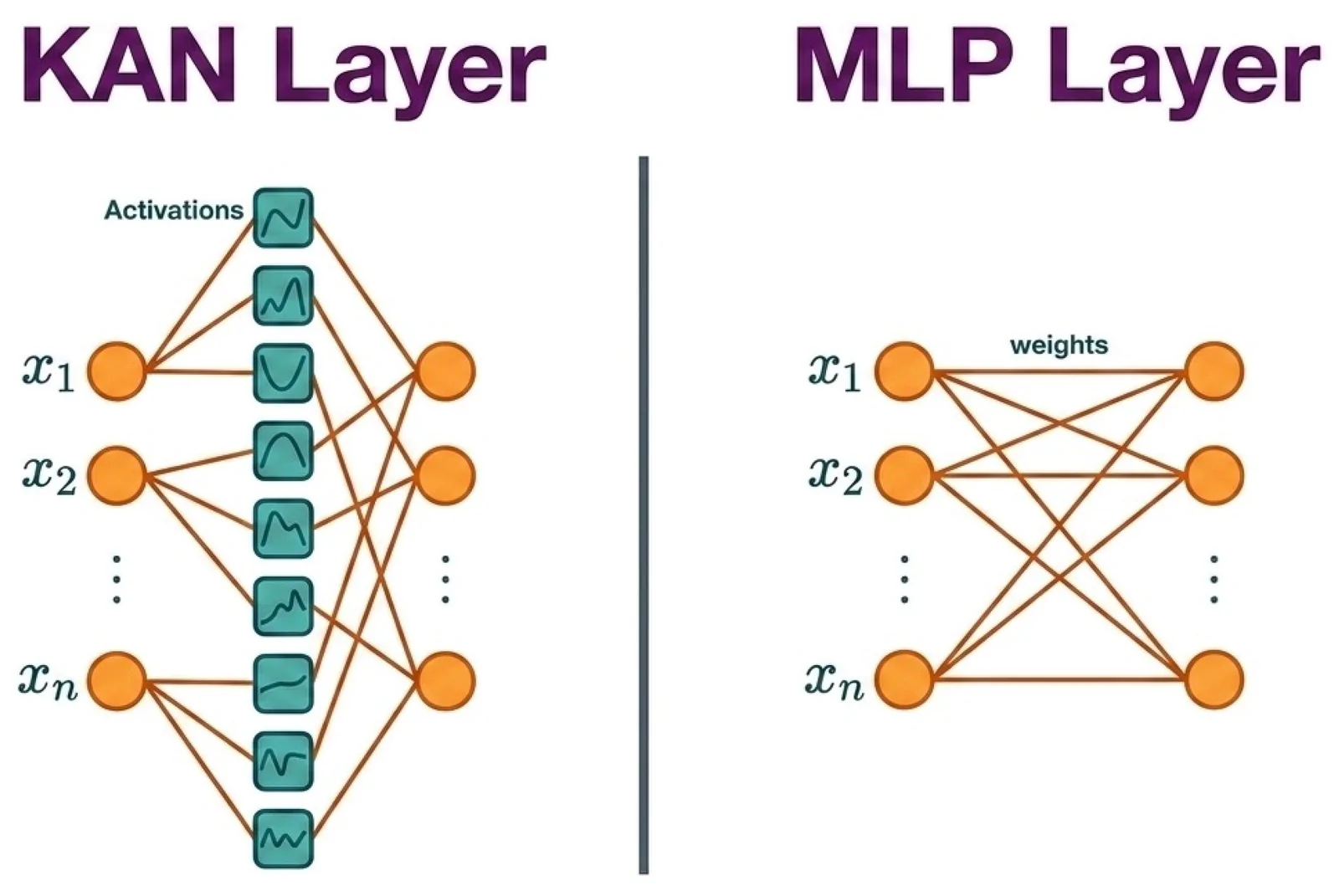
Schematic comparison of KAN and conventional MLP transformations.

This formulation enables a richer characterization of complex interactions among neighboring atoms and local chemical environments. To clarify the architectural distinction between the conventional MLP transformation and the KAN-based transformation used in KAN-GIN, Figure 7 provides a schematic comparison of the two nonlinear mapping mechanisms. In a conventional MLP layer, learnable scalar weights are associated with the connections between neurons, and nonlinear activation functions are applied at the nodes. In contrast, the KAN formulation replaces these fixed scalar-weight connections with learnable univariate nonlinear functions defined along the edges. In KAN-GIN, this KAN-based transformation replaces the post-aggregation MLP, while the remaining message-passing framework is kept unchanged.

For completeness, and to facilitate reproducibility, the complete forward propagation procedure of KAN-GIN is summarized in Algorithm 1. The algorithm follows the notation introduced above. At each graph layer, the representation of node *v* is combined with the representations of its neighbors according to the GIN aggregation operator, after which the conventional MLP transformation is replaced by the KAN transformation. After *K* message-passing layers, global max pooling produces the molecular representation $h_{D}$. The independently encoded protein representation $h_{P}$ is concatenated with $h_{D}$, and the resulting joint representation $h=[h_{D};h_{P}]$ is passed to the fully connected prediction function f(·) to obtain the predicted affinity $\hat{y}$. The complete architecture and training hyperparameters used in the controlled experiments are provided in Table A1, including the molecular graph encoder, KAN configuration, protein encoder, fusion module, and training protocol.
**Algorithm 1** KAN-GIN forward propagation for drug-target affinity prediction**Require:** 
Molecular graph G=(V,E) with initial node representations ${\left\{h_{v}^{\left(0\right)}\right\}}_{v\in V}$; protein sequence $P=(a_{1},a_{2},\ldots ,a_{n})$; number of graph layers *K***Ensure:** 
Predicted drug-target affinity $\hat{y}$  1:**for** k=1 to *K* **do**  2:      **for** each node v∈V **do**  3:            $h_{v}^{\left(k\right)}\leftarrow {\mathrm{KAN}}^{\left(k\right)}\left(\left(1+\epsilon^{\left(k\right)}\right)h_{v}^{\left(k-1\right)}+\sum_{u\in N(v)}h_{u}^{\left(k-1\right)}\right)$  4:      **end for**  5:**end for**  6:$h_{G}\leftarrow \max_{v\in V}h_{v}^{\left(K\right)}$  7:$h_{D}\leftarrow h_{G}$  8:$h_{P}\leftarrow \mathrm{ProteinEncoder}\left(P\right)$  9:$h\leftarrow [h_{D};h_{P}]$10:$\hat{y}\leftarrow f\left(h\right)$11:**return** $\hat{y}$

#### 4.4.3. Graph-Level Representation

After multiple message-passing layers, node embeddings are aggregated through global max pooling:

(4) $$h_{G}=\max_{v\in V}h_{v}^{\left(K\right)}.$$

The resulting graph embedding summarizes the entire molecular structure and serves as the drug representation.

#### 4.4.4. Affinity Prediction

The drug embedding $h_{D}$ and protein embedding $h_{P}$ are concatenated:

(5) $$h=[h_{D};h_{P}].$$

The fused representation is passed through fully connected layers to predict the binding affinity score:

(6) $$\hat{y}=f\left(h\right).$$

The model is trained using the mean squared error (MSE) between the predicted and experimental affinity values.

### 4.5. Training Procedure

All neural-network models were implemented using PyTorch v2.11.0 with CUDA 12.8 and PyTorch Geometric v2.8.0.post1. The models were trained using the Adam optimizer.

Model selection was performed using validation performance only, and the held-out test subsets were not used for hyperparameter selection or checkpoint selection. Unless otherwise specified, training was limited to 80 epochs with early stopping, and the checkpoint corresponding to the minimum validation MSE was retained for final test-set evaluation.

**Principal Benchmark Protocol:** For the principal comparison reported in Table 1, GIN and KAN-GIN retained their respective model-specific configurations, which were held constant across DAVIS, KIBA, and METZ. GIN used a learning rate of $1.0\times 10^{-4}$ and weight decay of $1.0\times 10^{-5}$, whereas KAN-GIN used a learning rate of $1.5\times 10^{-4}$ and no weight decay. All results were obtained over three training seeds (42, 123, and 2025) and are reported as the mean ± standard deviation. MGraphDTA and GNPDTA were additionally included in the principal benchmark. Architecture-specific molecular and protein encoders were retained where required, while standardized interaction records, affinity-label transformations, train/validation/test assignments, checkpoint-selection principles, and evaluation functions were kept consistent across reproduced models. For GNPDTA, dataset-specific hyperparameter selection was performed using validation performance only; after selection, each configuration was fixed for the three reportable runs. Complete benchmark and training settings are provided in Appendix A.

**Controlled GIN-KAN/GIN Ablation:** In contrast to the principal benchmark, the controlled ablation used a single common configuration for both GIN and KAN-GIN across DAVIS, KIBA, and METZ. Hyperparameters were intentionally held fixed to evaluate the MLP-to-KAN architectural replacement under matched optimization conditions. The two models used identical preprocessing, random-interaction partitions, training seeds (42, 123, and 2025), protein encoder, fusion module, prediction head, optimization settings, and minimum-validation-MSE checkpoint-selection criterion. The complete architecture and training configuration for this controlled experiment is reported in Appendix A (Table A1).

### 4.6. Evaluation Metrics

Performance was evaluated using mean squared error (MSE), root-mean-square error (RMSE), mean absolute error (MAE), the coefficient of determination ($R^{2}$), and the concordance index (CI). Lower MSE, RMSE, and MAE values indicate lower prediction error, whereas higher $R^{2}$ and CI values indicate better predictive performance.

Separate from the principal benchmark comparison reported in Table 1, we conducted an additional fixed-split generalization experiment using split seed 2026. Four evaluation scenarios were considered: random interaction, cold drug, cold target, and double-cold. In the random-interaction protocol, standardized drug-target pairs were assigned to the training, validation, and test partitions while keeping duplicate instances of the same standardized pair within a single partition. In the cold-drug and cold-target protocols, drug and target identities, respectively, were partitioned such that the corresponding test entities were absent from training. In the double-cold protocol, drugs and targets were independently partitioned, and only interactions for which both entities belonged to the same partition were retained; cross-partition interactions were excluded.

For each dataset and evaluation scenario, GIN and KAN-GIN were trained and evaluated using exactly the same fixed partition. Training was repeated with seeds 42, 123, and 2025, checkpoint selection was based exclusively on the minimum validation MSE, and final performance was evaluated on the held-out test partition and reported as the mean ± standard deviation across the three training seeds. The random-interaction condition serves as an internal reference for this generalization experiment and is distinct from the principal benchmark reported in Table 1. Complete training settings and partition statistics for the four evaluation protocols are provided in Appendix A (Table A1 and Table A3).

### 4.7. Pair-Level Latent Representation Analysis

Beyond predictive performance, we investigated whether replacing the conventional MLP transformation with an adaptive KAN transformation influences the organization of the joint drug-target representation space. For each evaluated interaction, a joint representation was extracted from the penultimate fusion layer after combining the molecular graph and protein-sequence representations. Consequently, every latent vector corresponded to a specific drug target pair and its experimentally measured affinity.

**Local Affinity Predictability and Cross-Drug Local MAD:** For each evaluated drug-target interaction *i*, let $z_{i}$ denote the joint representation extracted from the penultimate fusion layer after combining the molecular graph and protein sequence representations, and let $y_{i}$ denote the corresponding observed pair-level affinity. Local affinity predictability was evaluated using a distance-weighted *k*-nearest-neighbor regressor with k=20. Out-of-fold predictions were generated using five-fold grouped cross-validation, with compound identity used as the grouping variable. Consequently, all interactions involving the same drug were assigned to the same fold. Within each fold, the embedding dimensions were standardized to zero mean and unit variance using only the fitting portion of that fold. For an interaction *i* in the evaluation fold, let $N_{20}^{\mathrm{train}}\left(i\right)$ denote its 20 nearest neighbors from the fitting fold under Euclidean distance. Its affinity was estimated as

(7) $${\hat{y}}_{i}^{\mathrm{kNN}}=\frac{\sum_{j\in N_{20}^{\mathrm{train}}\left(i\right)}w_{ij}y_{j}}{\sum_{j\in N_{20}^{\mathrm{train}}\left(i\right)}w_{ij}},\;w_{ij}=\frac{1}{d(z_{i},z_{j})+\epsilon },$$

where $d(z_{i},z_{j})$ is the Euclidean distance between the standardized joint representations and ϵ is a small constant used to avoid division by zero. Local affinity predictability was quantified using the coefficient of determination, $R^{2}$, between the observed affinities $y_{i}$ and their out-of-fold predictions ${\hat{y}}_{i}^{\mathrm{kNN}}$. Higher $R^{2}$ indicates greater local affinity predictability.

Local affinity consistency was evaluated using cross-drug Local Mean Absolute Deviation (Local MAD). For each interaction *i*, let $N_{20}^{\neq d}\left(i\right)$ denote the set of its 20 nearest pair-level representations after excluding the query interaction and all interactions sharing its drug identity. Cross-drug Local MAD was defined as

(8) $$\mathrm{Local}\;\mathrm{MAD}=\frac{1}{N}\sum_{i=1}^{N}\left[\frac{1}{\left|N_{20}^{\neq d}\left(i\right)\right|}\sum_{j\in N_{20}^{\neq d}\left(i\right)}\left|y_{i}-y_{j}\right|\right],$$

where *N* is the number of evaluated drug-target interactions. Lower Local MAD indicates that nearby joint representations associated with different drugs have more similar observed affinity values. These analyses were conducted as post hoc probes of the organization of the joint drug-target representation space. The drug-grouped kNN procedure prevents compound-identity overlap within the auxiliary kNN folds but is distinct from the separately conducted cold-drug evaluation of the complete DTA model. The kNN regression, grouped cross-validation, and cluster-validity analyses were implemented using scikit-learn v1.9.0.

**Affinity-Bin Separability and Cluster Organization:** Each evaluated drug-target interaction was assigned to an affinity category according to its pair-specific experimentally measured affinity. The Davies-Bouldin (DB) and Calinski-Harabasz (CH) indices were calculated from the standardized joint drug-target representations. The DB index evaluates within-group compactness relative to between-group separation, with lower values indicating better organization. The CH index compares between-group dispersion with within-group dispersion, with higher values indicating better separation. These indices were compared between GIN and KAN-GIN within each dataset and were not compared directly across datasets.

### 4.8. Molecular Complexity Analysis

To examine whether the relative performance of KAN-GIN varies with molecular structural complexity, compounds were grouped into complexity bins using molecular descriptors, including molecular weight, number of atoms, topological polar surface area (TPSA), hydrogen-bond acceptor count, and number of rotatable bonds. For each molecule, prediction improvement was defined as the reduction in absolute prediction error achieved by KAN-GIN relative to the GIN baseline:

(9) $$\Delta E=\left|y-{\hat{y}}_{\mathrm{GIN}}\right|-\left|y-{\hat{y}}_{\mathrm{KAN}\text{-}\mathrm{GIN}}\right|,$$

where *y* denotes the true affinity value and $\hat{y}$ denotes the predicted affinity.

Molecules were subsequently divided into four complexity groups (small, medium, large, and very large) according to complexity score quantiles. Mean and median prediction improvements were computed within each group to evaluate whether the advantage of KAN-GIN varied with molecular complexity.

### 4.9. Saliency-Based Interpretability

To analyze the molecular patterns learned by the proposed model, gradient-based saliency maps were generated for selected drug compounds. The gradient of the predicted affinity with respect to atom features was computed, and the magnitude of these gradients was used as an importance score for each atom. Saliency values were normalized and projected onto the molecular graph to visualize chemically relevant regions contributing to affinity predictions. Representative structurally simple and structurally complex molecules were selected from the test set. Gradient-based saliency maps were generated for both GIN and KAN-GIN using the same attribution procedure to facilitate qualitative comparison of learned molecular representations.

#### 4.9.1. Molecular Docking

Molecular docking was performed using the CDOCKER protocol implemented in BIOVIA Discovery Studio v24.1.0.321712. The PRC2-EZH2 structure corresponding to PDB ID 5WG6 was used for structure-based assessment, with the EZH2 SET domain selected as the target region. Prior to docking, the protein structure was energy-minimized, and a receptor grid was generated around the binding cavity based on key amino-acid residues defined using the corresponding standard ligand. For each candidate compound, 10 docking poses were generated using the CDOCKER protocol. The resulting poses were evaluated according to the −CDOCKER Interaction Energy, with higher values used to select the preferred docked conformation. The selected protein-ligand complexes were subsequently examined using BIOVIA Discovery Studio to visualize the predicted binding poses and characterize ligand-protein interactions, including hydrogen-bond interactions. The −CDOCKER Interaction Energy is reported as the docking metric in the revised analysis. This quantity is distinguished from the separate binding-energy calculation generated during the original computational analysis and is not interpreted as an experimentally measured binding free energy.

#### 4.9.2. Candidate Screening and Compound Prioritization

An initial library of 1039 FDA-approved small molecules was considered for candidate screening. Compounds were filtered according to physicochemical and drug-likeness criteria, including molecular weight, Rule-of-Five properties, small-molecule status, and approved-drug status. Tazemetostat, an approved EZH2 inhibitor, was used as the reference compound for structural-similarity screening, resulting in 227 candidate molecules. Further database comparison and filtering reduced the candidate set to 51 compounds, which were subsequently evaluated according to ADME-related properties, including blood-brain barrier permeability, hydrogen-bond donor and acceptor counts, rotatable bonds, gastrointestinal absorption, and P-glycoprotein substrate status.

Prior to final candidate ranking, compounds were standardized to their parent molecular structures, and duplicate entries and alternative salt forms representing the same parent compound were consolidated. Candidate compounds were then evaluated using KAN-GIN affinity prediction followed by molecular docking for complementary structure-based assessment.

## 5. Conclusions

This study investigated whether replacing the conventional post-aggregation MLP transformation within a GIN molecular encoder with a KAN-based functional approximator can improve drug-target affinity prediction. The resulting KAN-GIN framework was evaluated on the DAVIS, KIBA, and METZ benchmarks through comparison with a matched GIN baseline and representative DTA architectures, controlled architectural ablation, entity-level generalization experiments, and analyses of the learned joint drug-target representations.

Across the principal benchmark, KAN-GIN consistently reduced prediction error relative to the matched GIN baseline, although performance relative to other DTA architectures was dataset-dependent. The controlled ablation further showed that the effect of replacing the conventional GIN transformation with the KAN-based transformation depends on the dataset and evaluation metric, supporting a more specific conclusion of potential predictive benefit rather than uniform superiority. Importantly, the additional cold-split experiments showed generally improved error-based generalization to unseen molecular entities, with the most consistent improvements observed for unseen drugs. These results provide evidence that the benefit of the KAN transformation is not restricted to random interaction splits in which molecular identities may be shared between the training and test sets.

Analysis of the joint drug-target representation space further indicated dataset-dependent differences in affinity-associated organization. KAN-GIN showed improved global affinity-bin organization on KIBA and METZ and the clearest improvement in local affinity organization on KIBA. These findings suggest that the nonlinear transformation can influence the geometry of the learned representation space; however, such computational organization should not be interpreted as evidence that the representations encode mechanistic pharmacology or biological causality.

The EZH2 drug-repurposing case study additionally demonstrated how KAN-GIN can be incorporated as an upstream candidate-prioritization component within a broader computational screening workflow. Molecular docking provided a complementary structure-based ranking rather than quantitative validation of the model predictions, as correspondence between the KAN-GIN and docking rankings was dataset- and scoring-function-dependent and did not reach statistical significance.

Several limitations remain. The magnitude of the KAN-GIN advantage varied across datasets, evaluation scenarios, and metrics, and the present experiments were restricted to three kinase-focused affinity benchmarks. The latent-space and attribution analyses are post hoc computational probes and do not establish biological mechanisms. Likewise, the EZH2 prioritization results have not been validated experimentally. Future work should therefore evaluate KAN-based graph transformations across larger and more diverse drug-target datasets, additional graph and protein encoders, and prospective screening settings. Experimental validation of prioritized compounds and further investigation of the learned representations will also be necessary to determine their biological and translational relevance.

Overall, the results identify KAN-based post-aggregation transformation as a promising architectural alternative for graph-based DTA prediction. Rather than establishing KAN-GIN as universally superior to existing approaches, the findings demonstrate that modifying the nonlinear transformation within a controlled graph-learning framework can yield meaningful, but dataset-dependent, improvements in affinity prediction and generalization.

## Figures and Tables

**Table 1:** Controlled comparison with representative DTA architectures. All reproduced models used the same standardized interaction records, affinity-label transformations, train/validation/test assignments, and evaluation functions. Architecture-specific input encoders were retained. Results are reported as the mean ± standard deviation across three training seeds (42, 123, and 2025). Downward arrows indicate that lower values are better, whereas upward arrows indicate that higher values are better.

Dataset	Model	MSE ↓	RMSE ↓	MAE ↓	$R^{2}$ ↑	CI ↑
DAVIS	GIN	0.2622±0.0087	0.5120±0.0085	0.3162±0.0128	0.6643±0.0107	0.8787±0.0051
	MGraphDTA	0.2630±0.0126	0.5127±0.0122	0.3195±0.0044	0.6629±0.0248	0.8699±0.0083
	GNPDTA	0.2410±0.0141	0.4908±0.0143	0.2975±0.0065	0.6910±0.0267	0.8812±0.0080
	KAN-GIN	0.2435±0.0134	0.4933±0.0135	0.2918±0.0172	0.6879±0.0253	0.8815±0.0087
KIBA	GIN	0.2342±0.0041	0.4839±0.0042	0.3190±0.0065	0.6663±0.0043	0.8270±0.0015
	MGraphDTA	0.1961±0.0033	0.4429±0.0037	0.2743±0.0084	0.7204±0.0100	0.8505±0.0033
	GNPDTA	0.2533±0.0453	0.5020±0.0440	0.3207±0.0213	0.6399±0.0564	0.8227±0.0128
	KAN-GIN	0.2163±0.0034	0.4651±0.0063	0.3068±0.0079	0.6991±0.0029	0.8407±0.0039
METZ	GIN	0.3594±0.0109	0.5995±0.0090	0.4555±0.0092	0.6064±0.0158	0.7874±0.0071
	MGraphDTA	0.3561±0.0411	0.5961±0.0341	0.4558±0.0307	0.6097±0.0494	0.7848±0.0157
	GNPDTA	0.3431±0.0075	0.5857±0.0064	0.4445±0.0045	0.6244±0.0009	0.7915±0.0018
	KAN-GIN	0.3155±0.0044	0.5617±0.0039	0.4269±0.0018	0.6545±0.0034	0.7997±0.0022

**Table 2:** Controlled ablation comparing the conventional GIN transformation with the KAN-based transformation in KAN-GIN. Results are reported as the mean ± standard deviation over three independent runs using seeds 42, 123, and 2025. All other architectural components, preprocessing, data partitions, and optimization settings were held fixed. Bold values indicate the better numerical mean between GIN and KAN-GIN within each dataset-protocol combination and metric.

Dataset	Model	MSE	RMSE	MAE	$R^{2}$	CI
DAVIS	GIN	0.2554±0.0081	0.5053±0.0080	0.3055±0.0124	0.6730±0.0087	0.8811±0.0040
	KAN-GIN	0.2467±0.0048	0.4966±0.0049	0.2918±0.0136	0.6840±0.0141	0.8756±0.0088
KIBA	GIN	0.2259±0.0054	0.4752±0.0057	0.3137±0.0051	0.6782±0.0032	0.8289±0.0023
	KAN-GIN	0.2135±0.0076	0.4620±0.0083	0.3005±0.0028	0.6969±0.0019	0.8349±0.0010
METZ	GIN	0.3459±0.0133	0.5880±0.0113	0.4477±0.0065	0.6214±0.0079	0.7935±0.0037
	KAN-GIN	0.3196±0.0104	0.5653±0.0092	0.4288±0.0055	0.6501±0.0043	0.7983±0.0022

**Table 3:** KAN-GIN predictions and molecular-docking results for the 10 unique compounds retained in the revised EZH2 case study. Compounds are ordered according to the primary structure-based ranking criterion, −CDOCKER Interaction Energy, for which higher values indicate more favorable interactions under the applied CDOCKER scoring convention.

Docking Rank	Compound	−CDOCKER Interaction Energy	DAVIS Predicted $pK_{d}$	KIBA Predicted Score	METZ Predicted $pK_{i}$	Calculated Binding Energy (kcal/mol)
1	Tirbanibulin	55.4425	5.063	11.411	5.403	−42.48
2	Perphenazine	48.7121	5.106	11.327	5.319	−54.79
3	Acetophenazine	45.6608	5.130	11.161	5.361	−43.74
4	Hydroxychloroquine	45.3161	5.209	11.400	5.618	−110.74
5	Larotrectinib sulfate	44.9253	5.007	11.282	5.561	−85.30
6	Cobimetinib	44.6449	4.885	11.266	5.422	−26.43
7	Carphenazine	43.8473	5.387	11.179	5.350	−52.69
8	Pramoxine	41.8076	5.213	10.852	5.378	−45.48
9	Quetiapine	41.5983	5.274	11.210	5.241	−46.55
10	Hydroxyzine hydrochloride	38.1454	5.243	11.240	5.277	−66.68

The calculated binding-energy values are reported as additional Discovery Studio-derived quantities and are not interpreted as experimental binding free energies. Candidate ranking in this table is based on −CDOCKER Interaction Energy.

**Table 4:** Correspondence between KAN-GIN candidate rankings and the primary molecular-docking ranking based on −CDOCKER Interaction Energy. Rank correlations were calculated across the 10 unique docked compounds.

Model	Spearman ρ	*p*	Kendall τ	*p*	Top-3	Top-5
DAVIS	−0.612	0.060	−0.422	0.108	0/3	1/5
KIBA	0.588	0.074	0.467	0.073	2/3	4/5
METZ	0.442	0.200	0.333	0.216	0/3	3/5

None of the rank correlations reached statistical significance at p<0.05. Top-*k* overlap denotes the number of compounds shared between the KAN-GIN and docking top-*k* candidate sets.

**Table 5:** Summary of benchmark datasets used in this study.

Dataset	Proteins	Compounds	Interactions	Metric	Range
DAVIS	442	68	30,056	$pK_{d}$	5.0–10.8
KIBA	229	2111	118,254	KIBA Score	0–17.2
METZ	170	1423	35,259	$pK_{i}$	4.0–11.1

## Data Availability

The DAVIS, KIBA, and METZ benchmark datasets analyzed in this study are publicly available from their original sources. The source code, data-preprocessing procedures, standardized split manifests, model implementations, experimental configurations, random seeds, and evaluation functions required to reproduce the KAN-GIN experiments have been released during peer review and are publicly available at https://github.com/ablabedoui1/KANGIN accessed on 20 August 2026. The molecular-docking data supporting the case study are available from the corresponding author upon request.

## Abbreviations

The following abbreviations are used in this manuscript:

CI	Concordance Index;
DTA	Drug-Target Affinity;
EZH2	Enhancer of Zeste Homolog 2;
KAN	Kolmogorov-Arnold Network;
GAT	Graph Attention Network;
GCN	Graph Convolutional Network;
GIN	Graph Isomorphism Network;
GNN	Graph Neural Network;
KAN-GIN	Kolmogorov-Arnold Graph Isomorphism Network;
MAE	Mean Absolute Error;
MLP	Multilayer Perceptron;
MSE	Mean Squared Error;
RMSE	Root-Mean-Square Error;
SMILES	Simplified Molecular Input Line Entry System;
TPSA	Topological Polar Surface Area;
DB	Davies-Bouldin;
CH	Calinski-Harabasz;
kNN	k-Nearest Neighbors;

## Appendix A

**Table A1:** Architecture and training hyperparameters used for KAN-GIN in the controlled experiments. Italicized rows denote architectural-component and training-protocol group headings.

Hyperparameter	Configuration
Molecular graph encoder	
Molecular representation	Molecular graph G=(V,E)
Node-feature dimension	36
Graph operator	GIN neighborhood aggregation
Number of graph layers (*K*)	5
Graph hidden dimension	128
Post-aggregation transformation	KAN-based transformation
KAN hidden dimension	128
KAN grid size	6
Node normalization	Batch normalization
Node activation	SiLU
Graph pooling	Global max pooling
Drug representation ($h_{D}$) dimension	128
Protein encoder	
Protein input	Integer-encoded sequence $P=(a_{1},\ldots ,a_{n})$
Protein vocabulary size	22
Maximum sequence length	1000 residues
Amino-acid embedding dimension	128
Protein encoder	Three-layer 1D CNN
CNN channels	32, 64, 96
CNN kernel size	8
Protein pooling	Adaptive global max pooling
Protein representation ($h_{P}$) dimension	128
Fusion and prediction	
Fusion operation	Concatenation, $h=[h_{D};h_{P}]$
Drug representation dimension	128
Protein representation dimension	128
Fused representation dimension	256
Prediction head	Fully connected layers
Dropout	0.1
Controlled training protocol	
Train/validation/test split	70%/15%/15%
Split type	Random interaction split
Batch size	128
Optimizer	Adam
Loss function	Mean squared error (MSE)
Learning rate	$1.5\times 10^{-4}$
Weight decay	0
Maximum epochs	80
Early-stopping patience	12
Checkpoint selection	Minimum validation MSE
Final evaluation	Held-out test set
Random seeds	42, 123, 2025

**Table A2:** Generalization performance of GIN and KAN-GIN under random-interaction, cold-drug, cold-target, and cold-drug-target (double-cold) evaluation protocols. Results are reported as the mean ± standard deviation across three independent training seeds (42, 123, and 2025) using fixed partitions generated with split seed 2026. Bold values indicate the better numerical mean within each dataset and metric. Downward arrows indicate that lower values are better, whereas upward arrows indicate that higher values are better.

Dataset	Protocol	Model	MSE ↓	RMSE ↓	MAE ↓	$R^{2}$ ↑	CI ↑
DAVIS	Random interaction	GIN	0.3144±0.0106	0.5606±0.0094	0.3420±0.0100	0.5950±0.0137	0.8682±0.0072
DAVIS	Random interaction	KAN-GIN	0.2956±0.0217	0.5435±0.0201	0.3209±0.0079	0.6192±0.0279	0.8682±0.0032
DAVIS	Cold-drug	GIN	0.7103±0.0392	0.8426±0.0233	0.5345±0.0213	−0.0647±0.0587	0.6640±0.0202
DAVIS	Cold-drug	KAN-GIN	0.6102±0.0164	0.7811±0.0105	0.5160±0.0202	0.0853±0.0245	0.6738±0.0215
DAVIS	Cold-target	GIN	0.6240±0.0291	0.7898±0.0184	0.4594±0.0053	0.2324±0.0358	0.7716±0.0071
DAVIS	Cold-target	KAN-GIN	0.6266±0.0602	0.7909±0.0383	0.4765±0.0371	0.2293±0.0741	0.7664±0.0327
DAVIS	Double-cold	GIN	0.6929±0.0342	0.8323±0.0207	0.5381±0.0283	−0.0884±0.0537	0.6051±0.0445
DAVIS	Double-cold	KAN-GIN	0.6223±0.0309	0.7887±0.0197	0.4978±0.0255	0.0225±0.0485	0.6052±0.0400
KIBA	Random interaction	GIN	0.2214±0.0045	0.4705±0.0047	0.3084±0.0049	0.6751±0.0065	0.8259±0.0030
KIBA	Random interaction	KAN-GIN	0.2153±0.0015	0.4640±0.0016	0.2990±0.0055	0.6840±0.0022	0.8342±0.0010
KIBA	Cold-drug	GIN	0.4949±0.0530	0.7028±0.0372	0.4690±0.0364	0.2880±0.0763	0.7210±0.0275
KIBA	Cold-drug	KAN-GIN	0.4190±0.0266	0.6471±0.0204	0.4241±0.0142	0.3972±0.0383	0.7534±0.0103
KIBA	Cold-target	GIN	0.4250±0.0401	0.6514±0.0304	0.4653±0.0224	0.3465±0.0617	0.7019±0.0180
KIBA	Cold-target	KAN-GIN	0.3761±0.0400	0.6127±0.0322	0.4669±0.0337	0.4217±0.0615	0.7285±0.0087
KIBA	Double-cold	GIN	0.5498±0.0432	0.7411±0.0289	0.4948±0.0090	0.0746±0.0728	0.6445±0.0038
KIBA	Double-cold	KAN-GIN	0.4957±0.0556	0.7033±0.0397	0.5224±0.0556	0.1656±0.0935	0.6610±0.0138
METZ	Random interaction	GIN	0.4012±0.0409	0.6329±0.0324	0.4820±0.0265	0.5588±0.0450	0.7745±0.0151
METZ	Random interaction	KAN-GIN	0.3310±0.0049	0.5753±0.0042	0.4367±0.0030	0.6360±0.0054	0.7936±0.0029
METZ	Cold-drug	GIN	0.5909±0.0370	0.7684±0.0243	0.5864±0.0112	0.3811±0.0388	0.7315±0.0073
METZ	Cold-drug	KAN-GIN	0.5232±0.0299	0.7232±0.0209	0.5512±0.0161	0.4519±0.0314	0.7475±0.0081
METZ	Cold-target	GIN	0.7066±0.0501	0.8403±0.0295	0.6401±0.0214	0.2243±0.0550	0.7083±0.0028
METZ	Cold-target	KAN-GIN	0.5789±0.0154	0.7608±0.0101	0.5985±0.0089	0.3645±0.0169	0.7029±0.0060
METZ	Double-cold	GIN	0.8809±0.0921	0.9377±0.0484	0.7067±0.0352	0.0218±0.1022	0.6414±0.0207
METZ	Double-cold	KAN-GIN	0.6867±0.0259	0.8286±0.0156	0.6459±0.0190	0.2375±0.0288	0.6676±0.0144

Random interaction evaluates unseen interaction records while allowing drug and target identities to occur across partitions. Cold-drug evaluation holds out drug identities from training, cold-target evaluation holds out target identities, and double-cold evaluation requires both the drug and target of each test interaction to be absent from training. Bold values indicate the better mean performance between GIN and KAN-GIN within each dataset-protocol combination.

**Table A3:** Interaction counts for the separate fixed split-seed-2026 generalization experiment. Random-interaction partitions were constructed at the standardized drug-target-pair level. Cold-drug and cold-target partitions enforced disjoint drug and target identities, respectively. In the double-cold protocol, only interactions whose drug and target were assigned to the same partition were retained. Cross-partition interactions were excluded and were not used for training, model selection, or testing.

Dataset	Evaluation Scenario	Training	Validation	Test	Excluded
DAVIS	Random interaction	21,104	4427	4525	0
DAVIS	Cold-drug	21,216	4420	4420	0
DAVIS	Cold-target	19,924	5508	4624	0
DAVIS	Double-cold	15,456	600	600	13,400
KIBA	Random interaction	82,771	17,737	17,746	0
KIBA	Cold-drug	83,900	16,584	17,770	0
KIBA	Cold-target	80,140	19,939	18,175	0
KIBA	Double-cold	57,969	2413	2885	54,987
METZ	Random interaction	24,683	5288	5288	0
METZ	Cold-drug	24,592	5221	5446	0
METZ	Cold-target	25,185	5850	4224	0
METZ	Double-cold	16,900	949	747	16,663

**Table A4:** Training-set overlap and structural-similarity analysis of shortlisted compounds. Shortlisted compounds were standardized to their parent molecular structures and compared with compounds in the DAVIS, KIBA, and METZ benchmark datasets. Structural similarity was assessed using Morgan fingerprints (radius = 2, 2048 bits) and the Tanimoto coefficient. A Tanimoto similarity of ≥0.80 was used as an operational threshold for flagging close structural analogs. No exact structural matches or close analogs above this threshold were identified.

Candidate	DAVIS	KIBA	METZ	Exact	Close Analog
	Max. Sim.	Max. Sim.	Max. Sim.	Match	(≥0.80)
Hydroxychloroquine	0.284	0.333	0.333	No	No
Larotrectinib sulfate	0.172	0.256	0.256	No	No
Hydroxyzine hydrochloride	0.205	0.203	0.211	No	No
Perphenazine	0.244	0.227	0.324	No	No
Carphenazine	0.242	0.279	0.253	No	No
Quetiapine	0.209	0.228	0.213	No	No
Pramoxine	0.364	0.404	0.364	No	No
Acetophenazine	0.247	0.288	0.260	No	No
Tirbanibulin	0.323	0.427	0.345	No	No
Cobimetinib	0.403	0.325	0.438	No	No

**Table A5:** Sensitivity analysis of rank correspondence between KAN-GIN predictions and alternative docking-derived quantities. Spearman’s ρ and Kendall’s τ were calculated across the 10 unique docked compounds.

KAN-GIN Model	Docking Quantity	Spearman ρ	*p*	Kendall τ	*p*	*n*
DAVIS	−CDOCKER Energy	−0.055	0.881	−0.067	0.862	10
DAVIS	−CDOCKER Interaction Energy	−0.612	0.060	−0.422	0.108	10
DAVIS	Calculated Binding Energy	0.273	0.446	0.244	0.381	10
KIBA	−CDOCKER Energy	0.164	0.651	0.111	0.727	10
KIBA	−CDOCKER Interaction Energy	0.588	0.074	0.467	0.073	10
KIBA	Calculated Binding Energy	0.248	0.489	0.244	0.381	10
METZ	−CDOCKER Energy	0.297	0.405	0.244	0.381	10
METZ	−CDOCKER Interaction Energy	0.442	0.200	0.333	0.216	10
METZ	Calculated Binding Energy	0.067	0.855	−0.067	0.862	10

The sensitivity analysis was performed to determine whether conclusions regarding model-docking correspondence depended on the docking-derived quantity considered. None of the reported rank correlations reached statistical significance at p<0.05.
